# Application of Antioxidant Poly-Lactic Acid/Polyhydroxybutyrate (PLA/PHB) Films with Rice Bran Extract for the Preservation of Fresh Pork Meat

**DOI:** 10.3390/foods13060972

**Published:** 2024-03-21

**Authors:** María Cabeza de Vaca, María Rosario Ramírez-Bernabé, David Tejerina Barrado, Javier Rocha Pimienta, Jonathan Delgado-Adámez

**Affiliations:** Instituto Tecnológico Agroalimentario (INTAEX), Centro de Investigaciones Científicas y Tecnológicas de Extremadura (CICYTEX), 06187 Badajoz, Spain; maria.cabeza@juntaex.es (M.C.d.V.); david.tejerina@juntaex.es (D.T.B.); javier.rocha@juntaex.es (J.R.P.); jonathan.delgado@juntaex.es (J.D.-A.)

**Keywords:** antioxidant film, poly-lactic acid (PLA), active biodegradable packaging, meat preservation, rice bran extract (RBE)

## Abstract

Poly-lactic acid/polyhydroxybutyrate (PLA/PHB) bio-based films suppose an environmentally friendly alternative to petroleum-derived packaging. In addition, rice bran extracts (RBEs) are an interesting source of bioactive compounds. In the present study, active films were formulated with 0.3% (*w*/*v*) or 0.5% (*w*/*v*) RBE (low-RBE and high-RBE) and compared to PLA/PHB films with no RBE. The migrations of active compounds as well as the antimicrobial and the antioxidant activities were analyzed in the three film formulations. The effects of active PLA/PHB films on fresh pork meat were evaluated by measuring the instrumental color, lipid and protein oxidations, and microbiological status of meat refrigerated for 1, 5, or 9 days. The developed films presented antioxidant activity in vitro, but they did not have an antimicrobial effect against bacterial development (*E. coli* nor *L. innocua*). The PLA/PHB film with no extract prevented changes in the instrumental color of meat during storage. However, the antioxidant effect of the PLA/PHB films on fresh pork was negligible, and the inclusion of high doses of extract favored microbial development in the pork during storage. Despite the lack of activity of active PLA/PHB films on meat, their use could be a sustainable alternative to the petroleum-based films.

## 1. Introduction

Fresh meat products are highly perishable foods that are easily susceptible to spoilage by microbial growth and physicochemical oxidations. Both factors suppose a loss of health safety and chemical changes that involve the degradation of products. The protection of meat products from environmental factors is one of the main strategies for extending their shelf-life. Concretely, the use of surface packaging films, acting as oxygen and water vapor barriers, is one common technique to improve meat preservation. The addition of bioactive compounds with antimicrobial and antioxidant activities to packaging films can also improve their preservative function due to migration of the active agents to the headspace or onto the food surface [1,2,3]. This combination of films with active additives supposes the generation of active films, with a great interest in food preservation and the packaging industry.

Nowadays, most of the developed films are formulated with non-biodegradable plastics derived from fossil fuels, which entails negative environmental impacts [4]. The rapid increase in the use of plastic materials, together with greater social awareness of environmental and economic sustainability, makes the replacement of plastics derived from petroleum by biodegradable materials one of the challenges in European policy, being one aim to achieve sustainability. In this sense, the substitution of conventional films by biobased biopolymers that are derived from biological resources and are biodegradable results in an interesting, environmentally friendly alternative for the food packaging industry.

Among the several eco-friendly materials, poly-lactic acid (PLA)-biobased polymers have been shown to be an interesting substitute for conventional plastics due to their adequate properties for film formation and high biodegradability [5]. Moreover, PLA-based films present a series of adequate physical properties, like their ability to provide a good barrier to oxygen and water vapor, which makes them idoneous for food packaging [6]. Concretely, the effectiveness of PLA films to improve meat conservation has been demonstrated [2]. These characteristics, together with its possible obtention from biological resources on an industrial scale throughout the fermentation of agro-industrial by-products [7], provide PLA-based films with a special interest, both for the economic and environmental sustainability and for the added value that it provides to agro-industrial wastes. However, to improve the physical and technical properties of bio-based films, the addition of plasticizer or active additives is usually required, which can lead to the release of toxic compounds during the degradation of biopolymers [8]. For this reason, the replacement of chemical additives with others of natural origins is especially interesting. This is the case for PHB poly(hydroxybutyrate), a biodegradable thermoplastic polymer with a high crystallinity [4]. Thus, formulations of PLA plastics with polyhydroxybutyrate (PHB) can result an environmentally friendly and healthy solution to improve the PLA film properties due to its natural origin, high biodegradability, and adequate aptitude as a plastic material [4].

On the other hand, the integration of natural active additives, such as antioxidant or antimicrobial agents, is common to enhance the preservative properties of biofilms [1,9,10,11,12,13]. Thus, the application of active PLA/PHB films has been previously studied for meat preservation with positive results, improving the fresh meat conservation and extending its shelf-life [2,9]. Among the sources of bioactive compounds, rice bran constitutes a cheap agro-industrial by-product without a direct economic value but with a high content of natural lipophilic active compounds like tocopherols and γ-oryzanol. Among these compounds, γ-oryzanol is the main and most active compound linked rice bran extract (RBE), with a high yield in RBE [14]. γ-oryzanol is a mixture of ferulate esters of triterpene alcohol, where the major components are cycloartenyl ferulate, 24-methylene cycloartenyl ferulate, campestanyl ferulate, and sitosteryl ferulate [14]. RBE presents high antimicrobial and antioxidant activities and has also demonstrated adequate properties as a natural preservative in food, showing effective antimicrobial and antioxidant effects in fresh meat products [2,9,15,16,17,18,19]. Recently, an active conventional package (non-biodegradable) based on RBE was proposed to improve the conservation of sliced dry-cured Iberian ham, showing how it slowed down the growth of some microorganisms for up to 6 months while delaying the lipid oxidation and preventing discoloration during storage [16]. Based on these results, the implementation of new active films based on PLA/PHB biopolymers with RBE could be an attractive alternative to conventional plastic materials in order to improve the preservation of fresh meat due to their antioxidant and antimicrobial effects, along with the additional environmental interests for biofilms. 

However, variations in bio-films formulations can lead to modifications in the structural and physicochemical characteristics of the films, as well as changes in their effectivity [2,5,18]. Consequently, the effects of the addition of RBE on the properties of PLA biofilms need to be evaluated in depth. This is the first study that evaluates active PLA biofilms with RBE and their effectiveness for meat preservation. Therefore, the aim of the present study was to develop an active PLA/PHB bio-based film manufactured with rice bran extract (with antioxidant and antimicrobial properties) and to analyze its effectivity for the preservation of fresh pork during refrigerated storage.

## 2. Materials and Methods

### 2.1. Obtention of Rice Bran Extract

Bran rice was acquired from a local market and refrigerated (4 °C) in darkness. Its composition, according to the supplier, was as follows: moisture 10.0%, protein, 11.8%; carbohydrates, 19.0%; sugars, 3.9%; total fat, 19.4%; saturated fat, 3.9%; dietary fiber, 28.8%; and salt, 0.04%.

To obtain rice bran extracts (RBEs), extraction of the main bioactive compounds was carried out according to Martillanes et al. [14]. Thus, 100 mL of absolute ethanol was added to 10 g of dried milled bran rice in an Erlenmeyer flask. The mixture remained at 60 °C 97 min with shaking (Selecta, Unitronic OR model, Barcelona, Spain) in the dark. The extract solution was manually sieved and centrifuged at 10,000 rpm for 10 min at 4 °C, and the supernatant evaporated at 25 °C until it was completely dry. To characterize the obtained RBE, the quantification of γ-oryzanol content by HPLC (Agilent Technologies, Waldbronn, Germany), the determination of the antioxidant activity using the ABTS method, and the bacteriostatic activity were carried out according to Martillanes et al. [14]. The RBE had a yield of 8.37 mg γ-oryzanol g^−1^ extract and an antioxidant activity of 13.5 mg Trolox equivalent g^−1^ extract. For the antimicrobial activity, the minimum inhibitory concentration (MIC) resulted in a 6.62% of RBE for *E. coli* and 6.42% of RBE for *L. innocua*.

### 2.2. Biopolymer Films Formation

The biopolymer films were formed using a mixture of PLA, PHB (both of biological origin) and glycerol, that was previously dissolved in chloroform. Thus, 7.5% (*w*/*v*) of PLA was diluted in chloroform and completely solubilized using a magnetic stirrer (Selecta, Agimatic-N model, Barcelona, Spain) with heat. Afterwards, to improve the films’ crystallization, 2.5% (*w*/*v*) of PHB was added and stirred until solubilization. Subsequently, 1% (*w*/*v*) of glycerol was added and stirred to obtain a homogeneous solution. Finally, and according to the experiment, 0%, 0.3% (*w*/*v*), or 0.5% (*w*/*v*) of RBE (no RBE, low RBE, and high RBE) was incorporated into film solution and homogeneously diluted. Immediately, the film solutions were poured in 90 mm diameter petri dishes (22 g per dish) and left to dry at room temperature for 24–48 h in darkness, until complete desiccation and film formation occurred.

### 2.3. Migration Assay, Antioxidant Capacity, and Antimicrobial Characterization of Films

The RBE release assay was carried out according to the migration methods established in EU regulations (Commission regulation (EU) No. 10/2011) for simulants with high and intermediate polarities. The film migration of RBE was performed using two dilutions of standard food simulants: 95% ethanol (*v*/*v*) for fatty foods (D1) and 50% ethanol (*v*/*v*) (D2) for oil–water emulsions. A 10 × 15 mm^2^ piece of each film was immersed in 10 mL of the dilutions in sealed falcon tubes and kept at 30 °C with agitation in a shaker (Comecta, shaker D-2102 model, Barcelona, Spain) for 6, 12, 24, 48, 72, 144, or 240 h. After each time interval, the concentration of released RBE was determined by measuring the γ-oryzanol content using HPLC according to Martillanes et al. [14], employing cycloartenyl ferulate as the reference compound to quantify the all the compounds associated with γ-oryzanol. The results of the migrations were expressed as µg γ-oryzanol cm^−2^.

The total antioxidant capacity of the films was determined spectrophotometrically using a plate reader (Tecan, spark multimode microplate reader model, Grödig, Austry) by the ABTS radical scavenging method. A total of 19.2 mg of ABTS** was dissolved in 5 mL of persulfate 5 mM, diluted in ethanol 96% acidified with H_3_PO_4_, and stabilized for 1 h until a coloration level of 1.0 of absorbance at 734 nm was obtained. For the determination of the antioxidant activity, a 6 mm diameter portion of the film was directly added to the ABTS solutions. Measurements of films’ antioxidant capacities resulted from the decolorization of ABTS** for 2, 6, and 24 h. Inhibition of ABTS** radical cation was calculated using Trolox as the antioxidant standard and acidified ethanol as the blank. The results were expressed in µg Trolox equivalent cm^−2^.

Bacteriostatic activity of active PLA/PHB films for *Escherichia coli* (CECT 45) and *Listeria innocua* (CECT 910) was determined according to Jimenez et al. [18] with some modifications. Bacterial cultures stored at −80 °C were regenerated by transferring a loopful into Mueller–Hinton broth media and incubated at 37 °C for 20 h (Comecta, shaker D-2102 model, Barcelona, Spain) in order to obtain the cultures in the exponential phase of growth. Afterwards, fresh cultures were diluted into Mueller–Hinton broth media to obtain a target inoculum load of 10^6^ CFU (colony forming units) mL^−1^. Then, an aliquot of 100 µL was transferred to a tube with a 10 mL aliquot of Mueller–Hinton broth media. Film pieces (6 mm of diameter) of each formulation were placed in the inoculated tubes, with no film added for the control, then the samples were incubated at 37 °C for 24 h. Finally, the microbial counts were examined based on the culture of serial dilutions in petri dishes with standard plate count agar (PCA, Merck, Darmstadt, Germany) and incubated for 24 h at 37 °C. All the tests were performed in quadruplicate. To determine the inhibitory capacity of the PLA/PHB films, the loads for each culture (log CFU mL^−1^) were measured and the reduction of counts (%) with respect to the control (inoculum without film) were calculated.

### 2.4. Experimental Design and Packaging of Samples

Pork loins were purchased from a local butcher and sliced into 1 cm steaks. A total of 5 slices were used for the analysis of the proximate composition of the meat utilized in the experiments (Table 1). For the assay, in control steaks, no film was used, and the rest of steaks were covered in both surfaces with one of the three formulations of PLA/PHB films (no RBE, low RBE, or high RBE). All the meat samples were individually vacuum packaged (−0.8 bar) using Henkovac Proeco equipment (Henkovac International, Hertogenbosch, The Netherlands) in 10 × 10 cm^2^ polyamide polyethylene (20/100) bags of 120 μm thickness (OptiDure™ ODA7005, oxygen permeability: 10 cm^3^m^−2^, 24 h^−1^, and 0% relative humidity; Cryovac, Madrid, Spain). Finally, all the packaged samples were stored at 4 °C in darkness for 1, 5, or 9 days according to the experimental design. Once each storage time was reached, the films were removed for each sample. Immediately, the microbiological loads and instrumental color analysis were measured in the fresh steaks, whereas a portion of each steak was frozen at −80 °C for the determinations of the lipid and protein oxidations. For each combination of film formulation and storage time, a set of five steaks was analyzed. Therefore, a total of 60 loin steaks were evaluated (film type × replicates × storage time) (4 × 5 × 3).

### 2.5. Instrumental Color Parameters

The color was measured in duplicate for both sides of all the loin steaks once the films were removed. The measurements were performed using a Konica Minolta CM-5 Spectrophotometer (Konica Minolta, Tokyo, Japan). The reflectance was measured using an illuminant D65 and a viewing angle of 10°. A 30 mm diameter aperture was used. The color parameters were reported in relation to the CIELAB scale (L*, a*, and b*). The hue and chroma values were calculated using the following equations: Hue = atang(b*/a*); Chroma = √(a^2^ + b^2^). Thus, L* is related to lightness, a* is related to redness (+a*) or greenness (−a*), and b* indicates yellowness (+b*) or blueness (−b*), whereas the hue denotes the tone and chroma of the saturation. 

In order to detect color variations (ΔE), two types of comparisons were carried out. To quantify color deviations with respect to the original meat due to the film and time effects simultaneously, the coloration of each sample was compared to color of the control steaks on day 1 (ΔE_control_D1_). Subsequently, to determine the evolution due to conservation for each film, within a same film batch, the color evolution was contrasted with the corresponding samples on day 1 (ΔE_D1_). Therefore, the ΔE values were calculated using the following equations:

$${\Delta E}_{Control\_D1}=\sqrt{{\left(L^{*}-L_{control\_D1}^{*}\right)}^{2}+{\left(a^{*}-a_{control\_D1}^{*}\right)}^{2}+{\left(b^{*}-b_{control\_D1}^{*}\right)}^{2}}$$

$${\Delta E}_{D1}=\sqrt{{\left(L^{*}-L_{D1}^{*}\right)}^{2}+{\left(a^{*}-a_{D1}^{*}\right)}^{2}+{\left(b^{*}-b_{D1}^{*}\right)}^{2}}$$

### 2.6. Oxidative Status

The meat lipid oxidation was assessed according to the tiobarbituric reactive substances (TBA-RS) method [20] from previous extraction with trichloroacetic acid (TCA). A standard curve of malonaldehyde (MDA) was employed to calculate the TBA-RS, and the results were expressed as mg MDA kg^−1^ meat.

The determination of protein oxidation was carried out following the spectrophotometric method [21] by determining the ratio of carbonyls/protein in the meat. The carbonyl concentration in the protein was calculated by measuring the absorbance of 2,4-dinitrophenylhydrazine (DNPH) linked to the carbonyl groups in oxidized proteins using a molar absorption coefficient of absorption of 21.0 mM^−1^ cm^−1^ at 370 nm. The protein concentration was calculated at 280 nm, with bovine serum albumin (BSA) used as the standard. The results were expressed as nmol of carbonyls mg^−1^ protein.

### 2.7. Microbiological Analysis

Mesophiles, psychrotrophs, molds and yeasts, total coliform bacteria, *Escherichia coli*, and *Staphylococcus aureus* counts were measured during the refrigerated storage. Thus, under aseptic conditions, 10 g of meat was taken and homogenized with 90 mL of peptone water (Merck, 1.07043) in a laboratory blender (Comecta, Stomacher^®^ 400 Circulator model, Barcelona, Spain). To reach appropriate dilutions, serial decimal dilutions were performed in sterile peptone water. Finally, 1 mL of each sample was poured or 100 µL was spread onto total count or selective agar plates. The total psychrotrophs and mesophilic counts were obtained using a standard plate count agar (PCA) (Merck, Darmstadt, Germany) and incubated at 30 °C for 72 h or 6.5 °C for 7 days, respectively. The molds and yeasts were incubated in YGC agar (Merck, Darmstadt, Germany) at 25 °C for 6 days. The total coliforms and *Escherichia coli* were incubated on Chromocult^®^ agar (Merck, 1.10426) at 37 °C for 24–48 h. *Staphylococcus aureus* was cultivated on Baird–Parker agar (Merck, 1.05406) after incubation at 37 °C for 24–48 h. All the results were expressed as Log10 CFU g^−1^, being the detection limit of each microorganism of 10 CFU g^−1^, except for *S. aureus*, which was 100 CFU g^−1^. Finally, the presence of *Salmonella* spp. and *Listeria monocytogenes* was determined according to ISO 6579-1, 2017 [22] and ISO 11290-1, 2017 [23], respectively, from 25 g of meat.

### 2.8. Statistical Analysis

The results were shown as mean ± standard deviation for each designed batch. A two-way analysis of variance was performed to analyze the interactions between both factors: the type of film and the storage time. The film formulation and storage time effects were also analyzed using a one-way analysis of variance (ANOVA) applied twice, and a Tukey’s test was applied to compare the mean values when the ANOVA showed significant differences. The Pearson correlation coefficient between the antioxidant activity and the RBE migration was also calculated. SSPS software (IBM Corp. Released 2020. IBM SPSS Statistics for Windows, Version 27.0. IBM Corp, Armonk, NY, USA) was used for all the analyses. 

## 3. Results and Discussion

### 3.1. Migrations, Antioxidant Capacity, and Antimicrobial Activity of PLA/PHB Films

According to previous publications, RBE ethanolic extracts have a high antioxidant and antimicrobial activity linked mainly to the presence of the lipidic compounds associated with γ-oryzanol [14,24]. For this reason, in active PLA/PHB films, the determination of their capacity to release oryzanol from the PLA/PHB net and its possible migration to the food matrix, as well as its final antioxidant and antimicrobial properties, becomes relevant when RBE is added.

The RBE migrations, measured as γ-oryzanol released, were carried out in two types of food simulants: ethanol 95% (D1) and ethanol 50% (D2). For simulant D2, no migration of γ-oryzanol was detected in any sample. For stimulant D1, a fast release was observed at the initial time, detecting the fastest diffusivities between 2 and 6 h (Table 2). From that moment, the migrations showed a constant increase throughout the first 24 h for the low-RBE level and 48 h for the high-RBE level, reaching the maximum release, an equilibrium with the solvent, at these moments. After these times, the migrations maintained similar levels until the end of the assay. The release behaviors were similar for both RBE levels, with no significant differences between the two doses at any of the sampling times. In general, the migration trend for D1 was in line with the referenced literature, since the highest release of active compounds from PLA was observed at 24–48 h [11,12,25]. Regarding the lack of migration in D2, similar results were observed by Gavril et al. [26] for compounds in lemon balm and sage powered added to PLA films, detecting migrations of no volatile compounds only when 95% ethanol was used. Furthermore, according to Ma et al. [25], the diffusion of cinnamaldehyde added to PLA/PHB films in several ethanolic dissolvents (100% water, 3% acetic acid, 10% ethanol, and 65% ethanol solutions) was only effective for the 65% ethanol simulant. Across all the conditions, no detection of γ-oryzanol in D2 was expected. This lack of migrations in D2 could be explained, in part, by the extraction temperature of the present assay (30 °C), since temperatures lower than 30 °C significantly slow down and reduce the liberation of certain compounds trapped in the PLA/PHB structure like chatequin [12] or tocopherol [11]. In this way, application of higher temperatures may enhance the release of RBE compounds in the D2 assay. On the other hand, the good capacity of ethanol to penetrate the PLA/PHB net and dilute active compounds is reduced by the addition of water. Therefore, a decline in ethanol content could significantly affect the migration kinetics, decreasing the release of active compounds from PLA/PHB [12,13]. These facts, together with the limited solubility of γ-oryzanol in aqueous solvents due to its lipophilic character, could explain the lack of detection of this compound in samples extracted using simulant D2.

**Figure 1:**
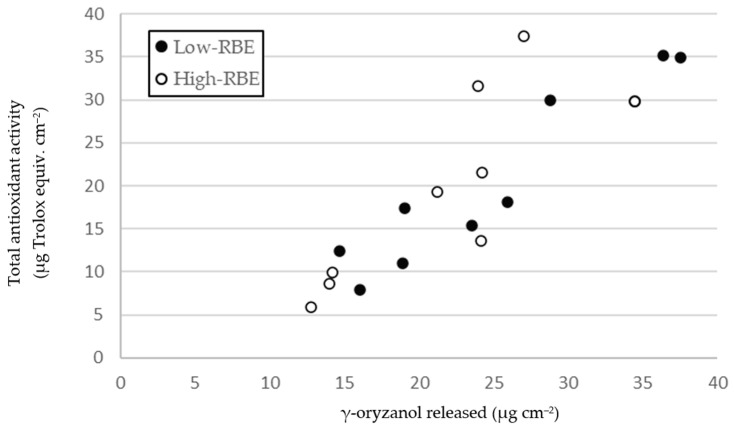
Correlation between total antioxidant capacity (ABTS) and RBE migrations in PLA/PHB films. Pearson’s correlation (coefficient = 0.867, significance ≤ 0.001).

The total antioxidant activity of PLA/PHB films with low and high doses of RBE over 24 h is shown in Table 2. Both films showed a continuous enhancement in ABTS radical scavenging at 24 h, with a maximum rate of increase within the first 2 h, slowing down at 6 and 24 h. Regarding the effects of the RBE dose, no significant differences in antioxidant capacity were observed between the low and high RBE concentrations. Comparing these values of the antioxidant activity of the films with the migration results of D1, the behaviors of both variables within the first 24 h were similar (Figure 1), and a positive correlation response between the γ-oryzanol released and the total antioxidant was observed (Pearson’s correlation coefficients r = 0.867, significant at *p* ≤ 0.001).

These results are in line with previous studies in which the addition of active compounds provided antioxidant properties to PLA/PHB films, reaching a maximum activity in the first hours [26,27]. Regarding the active compound dose, according to bibliography the higher incorporation of additives provides a higher antioxidant activity in films in most cases, especially when it comes from pure active compounds [28], but also from active complex extracts [26,27]. But this does not always occur. For certain extracts, an increment in concentrations only produces effective results from a minimum value and until to a determinate dose. In this sense, Llana-Ruiz-Cabello et al. [29] showed that the addition of 2% oregano essential oil into PLA films did not result in effective changes, and antioxidant activity was only detected from 5%, keeping similar values until levels of 10%. On the other hand, the effects on the antioxidant activity (ABTS and DPPH) were not observed in the case of *Allium* spp. extracts added to PLA films for doses of 2.5% and 6.5% [30]. These results could be explained by the changes produced on the physical and mechanical properties of the PLA/PHB films due to the inclusion of extracts [5], which subsequently modified the capacity of release compounds, as well as the nature [12] and quantity [9] of the active compounds added to the films. For this reason, a higher concentration of active compounds in PLA/PHB films does not always imply a higher release of active compounds nor an increase in antioxidants properties, as was observed in the present assay for RBE.

Concerning the results of the bacteriostatic activity of active films, inhibition of *E. coli* and *L. innocua* was not detected for any formulation According to the literature, neither PLA nor PHB films showed antimicrobial properties per se [10,31], so the antimicrobial properties of active PLA/PHB films could take place only when active compounds were added. On the other hand, the results of the bacteriostatic assays of active PLA/PHB films may depend on several factors, such as the initial antimicrobial activity of the added compounds, as well the type, origin, and purity of the extract or bioactive compounds [19,24,27,32], the final concentration of compound added, or the used microorganism [31]. Thus, some authors found antimicrobial responses of active films when active compounds were added to PLA or PLA/PHB films, but this only occurred when pure active compounds [28] or extracts with yields of active compounds of 82.15% (*v*/*v* essential oil) [33] were used. Overall, despite the antimicrobial activity observed in pure RBE against *E. coli* and *L. innocua*, no antimicrobial activity was linked to PLA films. This absence of antimicrobial activity of PLA/PHB films in vitro with RBE could be explained by the low yield of oryzanol in our RBE (8.37 mg γ-oryzanol g^−1^ extract) and by the final concentration of RBE extracts in the PLA/PHB films (5% *w*/*w* PLA for the highest levels), which would result an insufficient ability to hinder the growth of *E. coli* and *L. innocua* in active PLA. In line with these results, Llana-Ruiz-Cabello et al. [29] studied the effects of active PLA films enriched with oregano essential oil (2–10%) against several microorganisms, including *Escherichia coli*. At 24 h, these films only presented bacteriostatic activity for *Salmonella enterica* with 10% essential oil. They explained this behavior by the low yields of carvacrol and thymol detected in the final active PLA film. However, when the incubation periods were extended to 72 h, they observed a significant improvement in the antimicrobial properties of the active PLA films, being detected for 2, 5, and 10% essential oil concentrations against all the microorganism studied except for *E. coli*. Overall, for the present assay, an increase in RBE addition and longer periods of bacteriostatic assays could lead to an enhancement in the antimicrobial response of the active PLA films. 

On the other hand, our PLA/PHB films could present some effects against microbiological groups other than *E. coli* and *L. innocua* bacteria. In this respect, *Martillanes* et al. [15,19] reported that the addition of RBE reduced the microbial counts of molds and yeasts in mayonnaise-type emulsions and lactic acid bacteria in some meat products, like pork burgers treated by hydrostatic high pressure during storage. Similarly, an active conventional (non-biodegradable) packaging with RBE was effective in the preservation of sliced Iberian dry-cured ham, with the counts of mesophilic bacteria and molds and yeasts being reduced during refrigerated storage (at 4 °C for 180 days) [16]. Thus, the lack of antimicrobial activity against *E. coli* and *L. innocua* observed in vitro would not imply an absence of effects on the other microorganisms in the meat.

### 3.2. Effects of PLA/PHB Films with Rice Bran Extract on Fresh Pork Preservation

According to the referenced literature, the results of the proximate composition of the meat utilized in the present assay (Table 1) were within the normal ranges of pork loins [34,35].

The effects of the film treatment and time storage on the preservation of the pork loin steaks, as well as their interactions, were analyzed (Table 3). The effects of the type of film were only significant (*p* ≤ 0.05) with CIE L*, the counts of psychrotrophs, molds and yeasts, and *E. coli*. All the studied variables were significantly affected (*p* ≤ 0.05) by the time of storage except CIE a* and the total coliforms and *S. aureus* counts. Finally, a remarkable interaction crossing effect of film type and storage time was only observed for protein oxidation (*p* ≤ 0.01) and mesophile (*p* ≤ 0.01) and *E. coli* (*p* ≤ 0.05) counts. The independent effects of each factor are itemized in the tables below (Table 4 and Table 5).

The surface color of meat is one of the main factors in consumers’ choice of purchase and is used as an indirect indicator of quality. Thus, unexpected color variations can dissuade consumers from choosing a meat product [36]. In the current study, the values of the color parameters are shown in Table 4. The color of all the samples presented typical values, and normal evolutions linked to pork meat stored at refrigeration were observed [37,38].

In relation to the packaging, the effects of the film treatments were only observed on day 1 in CIE b* and chroma. With respect to the control, on day 1, significant increases in CIE b* (*p* ≤ 0.01) were linked to the low-RBE and high-RBE films. For chroma, on day 1, higher values (*p* ≤ 0.05) were only observed for the high-RBE film. These changes could be associated with the yellowness of the RBE incorporated into the PLA/PHB film, which could turn the meat toward a yellowish color. In this sense, Martillanes et al. [15] also observed an increase in CIE L*, CIE b*, and chroma in pork minced meat when RBE was directly mixed with the meat. These results, in part, could explain the significant variations associated with the high-RBE film on day 1 observed in the present assay.

With respect to the effects of the time of storage, all the samples increased CIE L* across the 9 days. These increments were significant for the control (*p* ≤ 0.05), low-RBE (*p* ≤ 0.01), and high-RBE (*p* ≤ 0.001) batches, while the PLA films with no RBE preserved the lightness across all the storage times (*p* > 0.05). These different behaviors of the films agree with the results of the two-ways ANOVA (Table 3). CIE a* was not significantly (*p* > 0.05) affected by storage across any of the applied treatments. Regarding CIE b*, despite the increments observed throughout the storage in all the batches, significant results were only obtained in the control (*p* ≤ 0.01) and high-RBE (*p* ≤ 0.05) samples, reaching values of 10.05 and 10.04 on day 9, respectively. The chroma, or color saturation, values only significantly increased in the control batch (*p* ≤ 0.01) from 8.58 on day 1 to 10.32 at the end of the conservation period. In general, the hue, or global observed tone, was modified in all the batches but was only significantly affected (*p* ≤ 0.05) by time for the high-RBE group. These variations in both variables can be mainly due to the variations observed in CIE b*.

In order to globally evaluate the changes in the colorimetric parameters, ΔE_control_D1_ was calculated. Variations in this parameter imply changes in each batch against the control batch on day 1, which allows us to know the effect of the films on color. On day 1, these changes were significant (*p* ≤ 0.001) for all the treatments with PLA but were similar among the three films formulations. In this sense, the NBS (National Bureau of Standards) ranges in the total color variations (ΔE) based on visual perception are not noticeable (0–0.5), slightly noticeable (0.5–1.5), noticeable (1.5–3.0), well-visible (3.0–6.0), great difference (6.0–12.0), and very great difference (>12). Thus, the ΔE_control_D1_ values on day 1 were between 2.29 and 2.74 when PLA/PHB films were used, which could be noticeable the human eye and therefore affect to consumers’ shopping decisions. On day 5 and day 9, the application of the PLA/PHB films did not result in significant (*p* > 0.05) modifications in the ΔE_control_D1_ value with respect to the control samples, showing that the four treatments resulted in similar color variations. Throughout the storage period, the ΔE_control_D1_ increased constantly for all the treatments, being significant only for the high-RBE film (*p* ≤ 0.05). Thus, the ΔE_control_D1_ reached values between 1.95 and 3.06 on day 5 and from 4.01 to 5.49 on day 9. Therefore, these color variations were noticeable or well-visible.

On the other hand, the total color variations ΔE_D1_ were also calculated. This parameter evaluates global changes within each treatment with respect to the initial values on day 1. ΔE_D1_ increased during storage, and the values on days 5 and 9 were similar in all the groups. That is, the differences were not significant (*p* > 0.05) among the treatments. Thus, the ΔE_D1_ value reached on day 5 indicated noticeable total color variations (between 2.29 and 3.06), and on day 9, the color changes were well-visible (from 3.07 to 5.54). Overall, the films formulated using PLA/PHB did not damage the color of the fresh pork loin and helped to control the variations in the studied parameters in a way that increased of ΔE_control_D1_ and ΔE_D1_ on day 9 for the three films, producing similar results to the control batch.

The evolutions observed in the color parameters can be explained by physical–chemical modifications linked to physiologic processes in meat. Thus, lightness variation can be due to modifications in light scattering derived from muscle fiber elongation and variations in sarcoplasmic protein distribution as the meat ages. All these processes lead to changes in the selective light absorption and refractive index on the surface tissues, affecting the paleness or darkness of the meat [39]. On the other hand, color evolution toward brown discoloration, and consequently variations in chroma and hue, can be mainly explained by oxidation process of myoglobin, which produces discolorations during storage [40]. Additionally, in our case, the increases in the yellowness of the pork loin with the RBE films could be explained by the contact of extract from the biofilms with the surface of the meat, given that the RBE had a yellow color. In this sense, a variation to a green tone and chroma was also observed by Martillanes et al. [19] in pork burgers when RBE was directly added.

The effects of active PLA or PHB films on the color of pork meat have been previously studied by several authors, and the results have been variable. In this sense, Hernández-García et al. [41] evaluated the effects of starch–gellan gum/PLA–PHB bilayer films with and without phenolic acids added (ferulic acid, *p*-coumaric acid, and protocatechuic acid) under similar conditions to our assay (at 5 °C for 15 days), and they found that the films containing phenolic acids preserved the color of the meat during storage. Freitas et al. [2,32] developed PLA films with rice straw fractions that were also effective in preserving the color changes. On the other hand, Martillanes et al. [16] showed that an active conventional package with RBE was effective in preventing discoloration during long storage times of Iberian sliced dry-cured ham. This differential effect of active films on meat color with respect to our results can be explained by the differences in formulations of the films and the composition of meat matrix. Thus, the addition of the extract could change the effectiveness of PLA/PHB films according to their chemical composition and the matrix nature. Thus, the positive effects showed by Hernández-García et al. [41] and Freitas et al. [2,32] in pork meat can be due to the aqueous nature of the extracts included in the films, whereas the RBE of the present assay was lipophilic. In the case of Martillanes et al. [16], the effectiveness of the RBE active film could be caused by the high fat content of Iberian dry-cured ham compared to the fresh commercial pork used in the current assay.

With regard to the antioxidant potential of active PLA/PHB in meat, in contrast to results obtained in vitro, the use of the PLA/PHB film had no effect on lipid oxidations on pork. Similar values of TBA-RS for all the film treatments (*p* > 0.05) were found on each day of storage (Table 4). In contrast to our results, Freitas et al. [2,32] showed that the application of PLA films with aqueous rice raw extracts resulted in effective control of lipid oxidation reactions in pork meat, and this effect was enhanced when higher concentrations of active extracts were added. These different behaviors could be explained by the type of extracts and the PLA formulation. Changes in films formulations can imply modifications in the properties of the films (like their oxygen and water permeability, light barrier capacity, or ability to release active compounds), and hence different effects on film effectiveness are expected.

In relation to the effects of storage, the samples showed an increase in lipid oxidation across the 9 days for all the film types. Overall, the TBA-RS values were like those previously obtained in pork meat [42] and in pork meat packaged with active PLA films [32]. Thus, for the present assay, the TBA-RS values started at values between 0.08 and 0.09 mg MDA kg^−1^ of meat on day 1 and reached values between 0.10 and 0.17 mg MDA kg^−1^ on day 9. Even so, the increments of TBA-RS over time were only significant for the control (*p* ≤ 0.05) and low-RBE film (*p* ≤ 0.01), keeping similar TBA-RS values (*p* > 0.05) throughout the 9 days in the no-RBE and high-RBE batches.

Application of PLA/PHB films resulted in a significant (*p* ≤ 0.001) control of protein oxidations on day 1, especially when the high-RBE dose was added (Table 4). The values at the beginning of the assay ranged from 1.06 (nmol carbonyls mg^−1^ protein) in the control samples to 0.74 (nmol carbonyls mg^−1^ protein) for the high-RBE film, with intermediate values for the no-RBE and low-RBE conditions. However, on day 5, the control presented lower values than the PLA/PHB films, and on day 9, the values were similar (*p* > 0.05) for the four treatments. Concerning the evolution throughout storage, only the control group presented similar values during storage, whereas for the samples with PLA/PHB films, the values trended to increase (*p* ≤ 0.05).

The literature regarding the implications of PLA or PHB films in protein oxidation of meat is scarce. One of the reasons can be the complexity of the mechanisms involved in protein oxidation. Protein oxidation is developed at three levels: the primary protein carbonylation derived from oxidative deamination of alkaline amino acids (lysine, threonine, arginine, and proline) by a radical-mediated mechanism; the secondary oxidation processes produced by deamination of alkaline amino acids throughout the Michael addition reactions from aldehydes or ketones, originating from lipid oxidations; and a third mechanism linked to oxidative cleavage of the peptide backbone via the α-amidation pathway or via oxidation of glutamyl side chains [43]. Despite this fact, Shahbazi and Shavisi [3] and Khezrian and Shahbazi [44], researching the extension of shelf-life on meat, studied the effects of chitosan- and cellulose-based films on protein oxidation in camel and beef meat. They found that the application of active films decreased the oxidative processes, and that the oxidative status of the meat was proportionally improved when active extracts were added. In the same way, Gallo et al. [45] found that the direct addition of *Echinacea angustifolia* extracts prevented lipid and protein oxidation in minced chicken meat. However, in the present study, the addition of RBE to PLA/PHB films did not improve the oxidation status during the refrigerated storage. Nevertheless, according to the literature, the antioxidant properties of RBE via direct application in meat have been demonstrated [19]. On the other hand, the of incorporation of active extracts in PLA and PLA/PHB films has been resulted effective to control the lipid oxidations in meat, both in pork [2,32,41] and beef [17]. These differences could be explained by the interactions between the PLA/PHB and the food matrix. The release kinetics of active compounds depend on the relaxation of polymeric matrix produced by the chemical interactions with the food matrix, which can be modified by the chemical affinity between the film and the food surface [1]. In this sense, the RBE migrations observed for PLA/PHB films in the present study (Table 2), especially the absence of release in simulant D2, could explain the lack of antioxidant effects on loin pork meat. Thus, the low content of fat in the analyzed meat (1.61%) makes this matrix comparable to the D2 simulant, in which migration of active γ-oryzanol linked to RBE was not detected. Consequently, for matrices with a hydrophilic character like loin pork meat, oryzanol migration may not occur. Moreover, considering that the storage of meat was carried out at 4 °C, a limited release of active compounds with antioxidant properties from the PLA/PHB films would be expected at that temperature. On the other hand, the oxygen and water permeability of PLA films can be increased by the addition of essential oil extracts [33] and other active compounds [6]. This effect could lead to an increase in the transmission of oxidant agents to the meat matrix, consequently counteracting the antioxidant activity of y-oryzanol released into the meat matrix. Therefore, a weak migration of RBE active compounds, together with a possible increase in oxygen permeability, could explain the lack of effect of the RBE from the PLA/PHB films on the oxidative status of the meat throughout the storage period.

The microbial counts of the pork with the PLA/PHB films during storage are shown in Table 5. Despite of the lack of activity of the films in vitro against the two pathogens analyzed (*E. coli* and *L. innocua*), the microbiological changes were evaluated on the pork loin steaks during the storage period, since some effects of the extract against other microbial groups could be produced, and this could affect the shelf-life of the meat products [16,19,29]. According to Codex Alimentarius, the meats of all the treatments were within their shelf-life until day 5, because on day 9, the populations of psychrotrophs microorganisms for all the groups were above the recommended levels. *Salmonella* spp. and *Listeria monocytogenes* were not detected on day 1 in any meat sample, and the *E. coli* loads throughout the study period were within the limits determined by European regulations (Commission Regulation (EC) No 2073/2005). In general, no marked differences were observed due to the utilization of PLA/PHB films or the addition of RBE. Hence, the application of PLA/PHB packaging did not improve the control of microbiological development in the pork meat. Furthermore, in comparison to the control batch, significant increases in mesophiles on day 5 (*p* ≤ 0.05) and psychrotrophs on day 9 (*p* ≤ 0.05) were observed when the highest RBE levels were added. Increases in molds and yeasts on day 1 (*p* ≤ 0.05) and total coliforms on day 5 (*p* ≤ 0.01) were also detected in the no-RBE films. In fact, the PLA/PHB packaging only showed a positive effect for *E. coli* loads on day 5 in the no-RBE group. The highest loads detected in the high-RBE film could be explained by the presence of nutrients in the extracts which could favor the proliferation of microbial populations and counteract the antimicrobial activity linked to γ-oryzanol. Overall, the development of active biopolymers based on the incorporation of pure active compounds derived from RBE, like γ-oryzanol, instead of the whole RBE extract could result an alternative, and could improve the antimicrobial properties of PLA/PHB films. The presence of *E. coli* and *S. aureus* was sporadic, being detected only in some samples and with loads too low to be considered significant (inferior to 30 total counts in agar culture). Thus, *E. coli* only appeared in some samples on day 1 of all the film treatments and on day 5 in all the batches except in no-RBE. At day 9, *E. coli* was not detected in any sample. The presence of *S. aureus* was detected on day 1 in all the treatments with PLA; on day 5 in some samples of the control, no-RBE, and high-RBE groups; and on day 9 in some samples of the no-RBE treatment.

In relation to the evolution during storage, mesophiles and psychrotrophs constantly increased in all the treatments. Molds and yeasts increased until day 5 (*p* ≤ 0.05) for the control and low-RBE groups, whereas the no-RBE and high-RBE groups showed similar values during all the storage time (*p* > 0.05). The counts of total coliforms were unchanged during storage (*p* > 0.05) for the four treatments. The *E. coli* counts decreased in the four treatments and were not detected on day 9 in any of the samples. The *S. aureus* loads decreased throughout the storage period, only being sporadically detected on day 9 in some samples of the no-RBE batch.

According to the literature, the effects of active PLA-based films on antimicrobial control in meat are variable. Some publications regarding the preservation of meat products demonstrate the effectiveness of incorporating certain active extracts into edible films [36,46], including PLA films [17]. For RBE, Martillanes et al. [19] did not find significant effects on day 1 on mesophilic, psychrotrophs, and lactic acid aerobic bacteria in pork burgers treated directly with RBE, although during storage, the burgers treated with hydrostatic high pressure and with RBE presented the lowest counts of lactic acid bacteria. However, in the present study, the addition of RBE to PLA/PHB did not improve the antimicrobial properties of the films on the meat matrix. In accordance with our results, no significant effects of the PLA/starch films without and with rice straw extracts have been previously reported for total viable counts, psychrotrophs bacteria, total coliforms, and lactic acid bacteria [2]. Other studies regarding the conservation of salad with active PLA films showed how the addition of oregano oil and allium commercial extracts in different concentrations only the reduced antimicrobial loads when the concentrations of active extracts in the PLA were higher than 5% [29,30]. Similarly, the total viable counts, total coliforms, and lactic acid bacteria with two types of phenolic acids were not reduced in pork packaged with bilayer PLA-based films [41]. This lack of antimicrobial response, both for pure PLA/PHB and active PLA/PHB with RBE, was expected, since no antimicrobial response against *Escherichia coli* and *Listeria innocua* was observed in vitro in the present study, and no migrations of RBE active compounds to the simulant D2, a matrix comparable to the analyzed meat, was detected in vitro. Furthermore, the antimicrobial activity of the film and in vivo migrations of the active compounds to the matrix do not always imply a reduction in the microbial loads in food [1]. However, in a previous study on Iberian sliced dry-cured ham conservation, a conventional (non-biodegradable) active packaging with RBE was effective against mold and yeast counts at 4 °C [16]. Unlike the meat in the present study (with 1.61% total fat), dry-cured ham is a fatty meat product. This could have favored the migrations of oryzanol from the package to this matrix, producing a slight antimicrobial effect.

## 4. Conclusions

The active PLA-based biofilms developed with different levels of rice bran extracts presented antioxidant activity in vitro, but they did not show an antimicrobial effect against the development of some pathogens (*E. coli* and *Listeria monocytogenes*). The antioxidant effects of the PLA/PHB biofilms on fresh pork were ineffective, and the use of high doses of extract could be detrimental for microbial control. However, the PLA films with no extract prevented the discoloration of steaks during storage. Therefore, the use of PLA films could be an alternative to petroleum-based films, with resulting environmental benefits. According to the in vitro results of the developed active films, they could be more suitable for the preservation of fatty foods, like sliced dry-cured meat products, which present a long shelf-life that is generally limited by the development of rancidness and oxidative reactions during storage. The development of bio-based films is a relatively recent challenge. Future studies should be focused on improving the formulations of active PLA biofilms in order to enhance the preservation of meat products according to nature of the matrix.

## Figures and Tables

**Table 1:** Physico-chemical compositon of fresh pork meat utilized in the assay.

pH	5.54 ± 0.03
Moisture (%)	73.20 ± 0.4
Protein (%)	24.07 ± 0.84
Total fat (%)	1.61 ± 0.29
Fatty acid profile (%):	
C12:0	0.10 ± 0.01
C14:0	1.38 ± 0.08
C16:0	23.77 ± 0.2
C16:1	3.65 ± 0.08
C17:0	0.38 ± 0.04
C17:1	0.34 ± 0.02
C18:0	11.52 ± 0.19
C18:1	44.85 ± 0.21
C18:2 (cis.cis)	12.94 ± 0.47
C18:3	0.45 ± 0.01
C20:1	0.63 ± 0.01

**Table 2:** Evolution of RBE in vitro release (γ-oryzanol ug cm^−2^) in simulant D1 (95% ethanol) and ABTS antioxidant activity (ug Trolox equiv. cm^−2^) of active PLA/PHB films enriched with RBE.

		Low-RBE	High-RBE	Significance
RBE release in D1	2 h	16.54 ± 2.15 c	13.72 ± 0.64 b	ns
	6 h	21.26 ± 4.24 bc	22.84 ± 1.59 ab	ns
	12 h	20.20 ± 1.70 bc	22.46 ± 4.89 ab	ns
	24 h	34.23 ± 4.75 a	27.64 ± 4.75 ab	ns
	48 h	29.13 ± 4.06 ab	32.91 ± 7.15 a	ns
	72 h	35.97 ± 6.08 a	31.15 ± 8.61 a	ns
	144 h	31.36 ± 1.88 ab	34.98 ± 3.20 a	ns
	240 h	35.50 ± 5.08 a	31.30 ± 9.66 a	ns
	Significance	***	***	
AAT	2 h	10.44 ± 2.28 a	8.1 ± 2.07 c	ns
	6 h	16.93 ± 1.42 b	18.18 ± 4.09 b	ns
	24 h	33.32 ± 2.95 c	32.97 ± 4.01 a	ns
	Significance	***	***	

Low-RBE: 0.3% rice extract added to film; High-RBE: 0.5% rice extract added to film. Results are expressed as MEAN ± SD. a, b, c: letters in the same column indicate statistically significant differences. Tukey’s test significance levels: ns—*p* > 0.05; ***—*p* ≤ 0.001.

**Table 3:** Two-way ANOVA. Effects of the type of film and storage on the preservation of pork loin fresh meat.

	Film	Time	Film × Time
Instrumental color			
CIE L*	**	***	ns
CIE a*	ns	ns	ns
CIE b*	ns	***	ns
Chroma	ns	***	ns
Hue	ns	*	ns
ΔE (control_D1)	ns	***	ns
ΔE (D1)	ns	***	ns
Lipid oxidation	ns	***	ns
Protein oxidation	ns	***	**
Mesophiles	ns	***	**
psychrotrophs	*	***	ns
Molds and yeasts	***	***	ns
Total coliforms	ns	ns	ns
*E. coli*	*	***	*
*S. aureus*	ns	ns	ns

Significance levels: ns—*p* > 0.05; *—*p* ≤ 0.05; **—*p* ≤ 0.01; ***—*p* ≤ 0.001.

**Table 4:** Instrumental color parameters, lipid oxidation (mg MDA kg^−1^ meat), and protein oxidation (nmol of carbonyls mg^−1^ protein) of pork loin steaks along the conservation period for each PLA/PHB film treatment.

	Storage Time	Control	No RBE	Low-RBE	High-RBE	Significance
CIE L*	Day 1	52.56 ± 2.13 x	53.53 ± 2.87	53.14 ± 2.32 x	50.79 ± 0.31 x	ns
	Day 5	53.20± 2.12 xy	54.51 ± 2.93	56.05 ± 1.46 y	52.81 ± 1.58 x	ns
	Day 9	55.98 ± 1.47 y	56.26 ± 1.67	57.79 ± 0.82 y	56.04 ± 1.75 y	ns
	Significance	*	ns	**	***	
CIE a*	Day 1	2.46 ± 1.02	2.58 ± 1.29	2.03 ± 0.94	3.28 ± 0.98	ns
	Day 5	2.52 ± 0.33	2.67 ± 1.00	1.94 ± 0.64	2.82 ± 0.55	ns
	Day 9	2.24 ± 0.82	1.91 ± 0.80	1.85 ± 0.49	2.05 ± 0.77	ns
	Significance	ns	ns	ns	ns	
CIE b*	Day 1	8.18 ± 0.44 bx	8.87 ± 0.22 ab	9.18 ± 0.41 a	8.93 ± 0.47 ax	**
	Day 5	9.13 ± 0.59 y	9.81 ± 1.04	9.75 ± 0.45	9.51 ± 0.61 xy	ns
	Day 9	10.05 ± 0.57 z	9.49 ± 0.43	9.67 ± 0.22	10.04 ± 0.61 y	ns
	Significance	**	ns	ns	*	
Chroma	Day 1	8.58 ± 0.61 bx	9.30 ± 0.35 ab	9.44 ± 0.38 ab	9.56 ± 0.53 a	*
	Day 5	9.48 ± 0.60 xy	9.98 ± 0.52	9.96 ± 0.45	9.93 ± 0.59	ns
	Day 9	10.32 ± 0.67 y	9.71 ± 0.38	9.86 ± 0.28	10.27 ± 0.54	ns
	*p*-value	**	ns	ns	ns	
Hue	Day 1	73.52 ± 6.25	73.99 ± 7.87	77.52 ± 5.71	69.99 ± 5.79 b	ns
	Day 5	74.55 ± 1.93	74.63 ± 6.81	78.79 ± 3.71	73.48 ± 3.23 ab	ns
	Day 9	77.94 ± 4.94	78.6 ± 4.87	79.24 ± 2.67	78.36 ± 4.5 a	ns
	Significance	ns	ns	ns	*	
ΔE (Control_D1)	Day 1	0.00 ± 0.00 bx	2.74 ± 1.54 a	2.48 ± 0.82 ax	2.29 ± 0.45 axy	***
	Day 5	2.29 ± 0.23 y	3.60 ± 1.62	3.91 ± 1.49 xy	1.95 ± 0.92 x	ns
	Day 9	4.01 ± 1.46 z	4.11 ± 1.47	5.49 ± 0.80 y	4.04 ± 1.82 y	ns
	Significance	***	ns	**	*	
ΔE (D1)	Day 1	0.00 ± 0.00 x	0.00 ± 0.00 x	0.00 ± 0.00 x	0.00 ± 0.00 x	
	Day 5	2.29 ± 0.23 y	3.00 ± 1.34 y	3.06 ± 1.42 y	2.39 ± 1.35 y	ns
	Day 9	4.01 ± 1.46 z	3.07 ± 1.47y	4.70 ±0.80 z	5.54 ± 1.88 z	ns
	Significance	***	**	**	***	
TBA-RS	Day 1	0.08 ± 0.01 x	0.09 ± 0.02	0.08 ± 0.03 x	0.09 ± 0.04	ns
	Day 5	0.13 ± 0.06 xy	0.19 ± 0.11	0.18 ± 0.06 y	0.17 ± 0.1	ns
	Day 9	0.16 ± 0.06 x	0.17 ± 0.07	0.10 ± 0.02 x	0.12 ± 0.07	ns
	Significance	*	ns	**	ns	
Protein Oxidation	Day 1	1.06 ± 0.11 a	0.92 ± 0.08 abx	0.89 ± 0.1 abx	0.74 ± 0.07 bx	***
	Day 5	0.83 ± 0.24 b	1.15 ± 0.12 ay	1.13 ± 0.15 ay	1.08 ± 0.05 aby	*
	Day 9	1.15 ± 0.25	1.17 ± 0.16 y	1.21 ± 0.11 y	1.27 ± 0.26 y	ns
	Significance	ns	*	**	**	

Control: without film; No RBE: no rice bran extract added to film; Low-RBE: 0.3% rice extract added to film; High-RBE: 0.5% rice extract added to film. Results are expressed as MEAN ± SD. a, b, c: different letters in the same row indicate statistically significant differences. x, y, z: different letters in the same column indicate statistically significant differences. Tukey’s test significance levels: ns—*p* > 0.05; *—*p* ≤ 0.05; **—*p* ≤ 0.01; ***—*p* ≤ 0.001.

**Table 5:** Microbiological changes (log CFU g^−1^) during storage of pork loin steaks with different PLA/PHB film treatments.

Microorganisms	Storage Time	Control	No RBE	Low-RBE	High-RBE	Significance
Mesophiles	Day 1	5.35 ± 0.25 x	5.55 ± 0.39 x	5.35 ± 0.19 x	5.09 ± 0.30 x	ns
	Day 5	5.78 ± 0.15 by	5.86 ± 0.3 abx	6.25 ± 0.26 aby	6.37 ± 0.40 ay	*
	Day 9	6.31 ± 0.22 z	6.58 ± 0.21 y	6.34 ± 0.35 y	6.40 ± 0.37 y	ns
	Significance	***	**	***	***	
Psychrotrophs	Day 1	5.10 ± 0.40 x	5.20 ± 0.29 x	5.17 ± 0.40 x	5.16 ± 0.23 x	ns
	Day 5	5.59 ± 0.47 x	5.56 ± 0.44 x	6.18 ± 0.46 y	6.38 ± 0.79 y	ns
	Day 9	7.01 ± 0.15 by	7.18 ± 0.13 aby	7.17 ± 0.33 abz	7.48 ± 0.3 az	*
	Significance	***	***	***	***	
Molds and yeasts	Day 1	3.56 ± 0.38 bx	4.32 ± 0.14 a	3.94 ± 0.20 abx	3.81 ± 0.31 ab	*
	Day 5	4.02 ± 0.17 y	4.35 ± 0.20	4.29 ± 0.13 y	4.20 ± 0.26	ns
	Day 9	4.13 ± 0.21 y	4.36 ± 0.29	4.04 ± 0.27 xy	4.11 ± 0.11	ns
	Significance	*	ns	*	ns	
Total coliforms	Day 1	3.02 ± 0.37	3.09 ± 0.42	2.95 ± 0.5	2.74 ± 0.33	ns
	Day 5	2.77 ± 0.46 b	3.37 ± 0.08 a	3.26 ± 0.15 ab	3.17 ± 0.35 ab	*
	Day 9	3.26 ± 0.38	2.94 ± 0.8	3.06 ± 0.73	3.22 ± 0.61	ns
	Significance	ns	ns	ns	ns	
*E. coli*	Day 1	1.74 ± 0.73 y	1.43 ± 0.66 y	1.28 ± 0.74 y	1.49 ± 0.74	ns
	Day 5	1.32 ± 0.82 ay	nd ± 0 bx	1.37 ± 0.57 ay	1.68 ± 0.69 ay	**
	Day 9	nd x	nd x	nd x	nd x	ns
	Significance	**	***	**	**	
*S. aureus*	Day 1	ND	2.06 ± 0.13	2.26 ± 0.29	2.06 ± 0.13	*
	Day 5	2.00 ± 0.00	2.12 ± 0.27	ND	2.06 ± 0.13	ns
	Day 9	ND	2.1 ± 0.21	ND	ND	ns
	Significance	ns	**	ns	ns	

Film effect: Control: without film; No RBE: no rice bran extract added to film; Low-RBE: 0.3% rice extract added to film; High-RBE: 0.5% rice extract added to film. Results are expressed as MEAN ± SD. nd and ND indicate that the results were below the detection limit of the method: nd < 1 log CFU g^−1^; ND < 2 log CFU g^−1^. a, b: different letters in the same row indicate statistically significant differences. x, y, z: different letters in the same column indicate statistically significant differences. Tuckey’s test significance levels: ns *p* > 0.05; * *p* ≤ 0.05; ** *p* ≤ 0.01; *** *p* ≤ 0.001. Abbreviations: CFU, colony forming units.

## Data Availability

The original contributions presented in the study are included in the article, further inquiries can be directed to the corresponding author.
